# Initiation and Regulation of Complement during Hemolytic Transfusion Reactions

**DOI:** 10.1155/2012/307093

**Published:** 2012-10-16

**Authors:** Sean R. Stowell, Anne M. Winkler, Cheryl L. Maier, C. Maridith Arthur, Nicole H. Smith, Kathryn R. Girard-Pierce, Richard D. Cummings, James C. Zimring, Jeanne E. Hendrickson

**Affiliations:** ^1^Department of Pathology and Laboratory Medicine, Emory University School of Medicine, Atlanta, GA 30322, USA; ^2^Department of Biochemistry, Emory University School of Medicine, Atlanta, GA 30322, USA; ^3^Department of Pediatrics, Aflac Cancer and Blood Disorders Center, Emory University, Atlanta, GA 30322, USA; ^4^Puget Sound Blood Center Research Institute, Seattle, WA 98102, USA

## Abstract

Hemolytic transfusion reactions represent one of the most common causes of transfusion-related mortality. Although many factors influence hemolytic transfusion reactions, complement activation represents one of the most common features associated with fatality. In this paper we will focus on the role of complement in initiating and regulating hemolytic transfusion reactions and will discuss potential strategies aimed at mitigating or favorably modulating complement during incompatible red blood cell transfusions.

## 1. Introduction

As pathogens began to evolve elaborate antigenic structures to avoid innate immunity, vertebrates evolved an equally impressive mechanism of combating antigenic diversity among pathogens [1]. Indeed, adaptive immunity appears to possess the capacity to respond to a nearly infinite number of antigens, enabling immunological protection against a wide variety of potential pathogens [1, 2]. However, not all foreign antigens represent a pathogenic threat. Although tolerance mechanisms exist that reduce the likelihood of developing antibodies against innocuous antigens, individuals can possess significant antibodies against antigenic polymorphisms on human tissue [2]. Indeed, hemolytic transfusion reactions typically reflect the engagement of antibodies directed against antithetical antigens on donor red blood cells (RBCs). 

The earliest example of human donor rejection occurred following transfusion of ABO (H) incompatible RBCs [3]. Although ABO(H) represent the first RBC polymorphic antigens described, many other carbohydrate and protein antigenic differences became apparent as transfusion practices increased [4]. Interestingly, these immune-mediated discoveries provided the first example of significant polymorphisms within the human population long before DNA was recognized as the molecular basis of inheritance [5–8]. As hemolytic transfusion reactions (HTRs) can occur following transfusion of incompatible RBCs or following transfer of antibodies present in donor units, such as platelets or plasma, significant testing occurs prior to transfusion to insure utilization of antigen compatible blood products [9, 10]. Unfortunately, these procedures occasionally fail. In addition, some patients fail to demonstrate detectable antibodies but exhibit amnestic antibody responses to previously exposed RBC antigens following transfusion [11]. Under these circumstances, cellular rejection in the form of a hemolytic transfusion reaction may occur. 

Hemolytic transfusion reactions may not only cause significant morbidity and compromise the therapeutic efficacy of transfusion, but ultimately these reactions can prove fatal. Indeed, hemolytic transfusion reactions represent one of the most common causes of transfusion-related mortality. Furthermore, in highly immunized patients, securing antigen compatible blood can be difficult, if not impossible, preventing appropriate and timely life-saving intervention [12]. As a result, a greater understanding of the factors that may influence hemolytic transfusion reactions is needed. Although many factors influence hemolytic transfusion reactions, in this paper we will focus on the potential role of complement in initiating and regulating hemolytic transfusion reactions, with a particular focus on potential strategies aimed at mitigating or favorably modulating complement during incompatible RBC transfusions. 

## 2. Early Transfusion Reactions

While many diseases reflect complement dysregulation [13], perhaps the earliest and most potent example of complement-mediated mortality predates the discovery of microbes and immunity. In 1667, Dr. Jean-Babtiste Deny transfused several patients multiple times with either sheep or calf blood. Although the patients appeared to initially tolerate transfusion, repeated transfusions uniformly resulted in patient death [4, 14]. Subsequent attempts nearly two centuries later utilizing human donors for transfusion resulted in more favorable outcomes; however, patients receiving transfusions from human donors occasionally experience similar fatalities despite many attempts to predict favorable responses to transfusion [4].

Prompted by previous work suggesting that antigenic differences on RBCs occur between different mammalian species, Karl Landsteiner sought to determine whether similar differences may account for incompatible transfusions using human donors [3]. In 1900, Landsteiner published his seminal work demonstrating that sera isolated from patients could differentially agglutinate donor RBCs [5]. Within the next decade, the discovery of A, B, and C (O) antigens enabled accurate prediction of immunological compatibility between donor and recipient, for which Landsteiner was awarded the Nobel prize in physiology and medicine in 1930 [3]. 

While the factors responsible for fatal outcomes during an incompatible transfusion remained unknown for many years, naturally occurring antibodies directed against carbohydrate xenoantigens on animal RBCs or ABO(H) antigens on human RBCs likely mediated activation of complement [15]. Robust complement activation not only results in significant intravascular hemolysis, but complement products also independently induce significant physiological changes. Indeed, early transfusion reactions likely reflected significant complement-mediated hemolysis and systemic alterations that ultimately resulted in fatal outcomes [15–18]. 

## 3. Complement: A Brief History

The identification of microbes as a potential cause of human illness drove intense research by numerous investigators to understand host factors that may inhibit microbial invasion. Early studies by Pastuer and others demonstrated that inoculation of animals with microbes could induce a form of host resistance to further infection [19]. Although the specific players responsible for acquired host immunity remained unknown for many years, both cellular and serological factors appeared to possess the ability to protect animals against reinfection [20]. 

Early studies focused on characterizing serological factors demonstrated that blood possesses intrinsic bactericidal activity following inoculation of a specific microbe. Subsequent studies demonstrated that heating serum at 55°C effectively eliminated this bactericidal activity *in vitro*. However, infusion of heat inactivated serum isolated from previously inoculated animals protected recipients from infection. These results suggested that serum contains heat labile and heat stable components. Paul Ehrlich subsequently coined the term complement to describe the heat labile component of immunity, which he postulated worked in concert with a heat stable “amboceptor” that provided target specificity [21]. Subsequent studies demonstrate that a complex pathway of complement activation and regulation occurs following “amboceptor” (antibody) engagement of antigen. 

## 4. Complement Initiation and Regulation: An Overview

**Figure 1:**
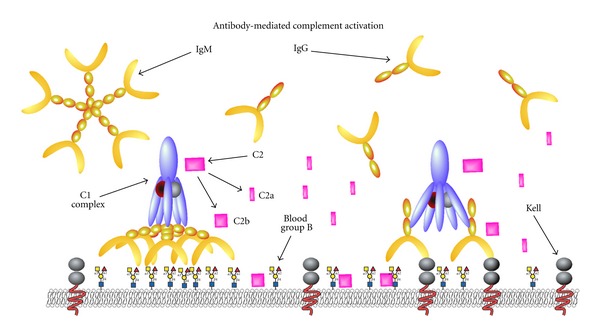
Antibody-mediated complement activation. IgM antibodies primarily exist as planar molecules. However, upon engagement of antigen, IgM antibodies undergo significant conformation changes that result in favorable exposure of multiple C1q binding domains. As a result, a single IgM molecule can initiate significant complement activation. In contrast, two separate IgG antibodies must engage antigen within close enough proximity to simultaneously engage the C1q fix complement.

Complement activation can be divided into three primary cascades of activation, classical (antibody), lectin, and alternative (Figure 1) [22, 23]. While the lectin and alternative pathways of complement activation play a key role in immunity [24, 25], antibodies provide the primary initiating activity of complement activation during a hemolytic transfusion [26]. Regardless of the initiating stimulus, each pathway converges on the formation of an enzyme complex capable of converting complement component C3 into active products, C3a and C3b. C3b, working in concert with additional complement components, ultimately propagates a cascade that terminates in the formation of a membrane attack complex and eventual target lysis [27].

Following transfusion of incompatible blood, antigen-antibody interactions facilitate engagement of the first component of the classical pathway, C1q. Once bound, C1q induces conformational changes in the serine protease, C1r, which allows C1r to cleave C1s, resulting in an active C1s protease [28]. This complex then cleaves C2 and C4, generating target bound C2b and C4b and the release of soluble C2a and C4a (Figure 1). Bound C2b and C4b form a complex, the C3 convertase, which cleaves C3 to form C3a and C3b [27]. C3a provides a soluble complement regulator of variety of biological pathways, including activation of mast cells, endothelial cells, and phagocytes in addition to intrinsically possessing antimicrobial activity [29, 30]. In contrast, C3b covalently attaches to the target membrane through a highly reactive thioester and thereby facilitates continuation of the complement cascade [23, 27]. Bound C3b, in complex with C2b and an additional C3b molecule, then facilitates conversion of C5 to C5a and C5b. Similar to C3a, C5a regulates a wide variety of systemic factors in immunity [31]. Bound C5b recruits additional complement factors, including C6, C7, and C8, which together facilitate the insertion and polymerization of C9 in the target membrane [32]. Ultimately, C9-mediated pore formation results in osmotic lysis of the target [32]. 

In contrast to the antibody-mediated pathway, the lectin and alternative pathways do not require adaptive immunity to target pathogen. For example, the lectin pathway utilizes a series of innate immune lectins, such as mannan binding lectin (MBL), which target microbes by recognizing common pathogen-associated molecular motifs [25]. Upon pathogen recognition, MBL engages adapter proteins, MASP-1 and MASP-2, which cleave C4 and C2 similar to C1s in the classical pathway [25, 28]. In contrast to the classic and lectin pathways, the alternative pathway does not actively engage pathogens through a target molecule. Instead, the alternative pathway relies on spontaneous activation of C3 to form C3 (H_2_O). C3 (H_2_O) then binds an additional complement factor, factor B, which renders factor B sensitive to cleavage by constitutively active factor D [24]. A complex of factor C3 (H_2_O) and Bb, stabilized by a factor P, then forms the C3 convertase of the alternative pathway [33, 34]. Importantly, C3b generated during antibody or lectin-induced activation can also directly facilitate D-mediated factor B activation, amplifying complement activation even when antibody alone can provide an initiating stimulus [25]. While antibody-antigen complexes primarily utilize the classic pathway, several studies demonstrate that antibody can also directly activate the alternative pathway [35]. As a result, although antibody-induced activation likely provides the primary driving force, each pathway likely contributes to the overall effect of complement-mediated transfusion reactions.

In addition to facilitating complement activation, complement factors, such as C3b, can serve as opsonins on the target membrane for a series of complement receptors [36]. Each complement receptor appears to be expressed on distinct leukocyte populations and tissue [37]. Furthermore, engagement of complement receptors on different cells can result in distinct immunological outcomes. For example, several complement receptors, including CR1 (CD35), CR3 (CD11bCD18), CR4 (CD11cCD18), and CRIg, typically facilitate phagocytosis of opsonized targets following C3b ligation [38–43]. In contrast, CR2 (CD21) engagement of complement can influence B cell activation and tolerance [44–46]. Additional complement receptors, which recognize soluble complement components, C3a and C5a, also regulate a wide variety of biological pathways following ligation [27, 31, 47]. Although not functionally important in complement activation, several complement factors actually possess unique antigenic structures used in blood group classification, such as the Cromer, Chido, and Rodgers blood groups, and therefore could also theoretically facilitate RBC removal as unique target antigens [48, 49]. Taken together, complement activation results in the production of a variety of highly active immunoregulatory components capable of influencing a broad range of biological processes. 

As with all immune effector functions, unregulated activation of complement can result in significant pathological sequelae. Indeed, many disease states are defined by genetic or acquired dysregulation of complement [50]. Given the evolutionary ancient history of complement, many inhibitory pathways exist that facilitate complement regulation. For example, constitutively expressed cell surface proteins, such as CD35 (CR1), CD46 (MCP), CD55, or CD59, provide various inhibitory activities to inhibit off-target complement activation [40, 51–53]. Importantly, each of these factors displays unique regulatory activities. For example, CD35 and CD46 bind C3b and C4b preventing complex formation with Bb and 2b, respectively, [40, 51, 54], thereby inhibiting active C3 convertase formation. Similarly, CD55 (decay accelerating factor or DAF) along with CD35 actively dissociates C3 convertase complexes once formed by displacing Bb and 2b [51, 53, 55]. Furthermore, CD59 (protectin) binds the C5, C6, C7, and C8 complex and inhibits C9 binding and formation of the membrane attack complex [56–58]. In concert, all of these mammalian, membrane bound receptors inhibit off-target complement deposition from actively destroying an individual's own cells. 

In addition to cell surface factors, several soluble factors also aid in complement regulation. Similar to the inhibitory activities of CD35, CD46, and CD55, C4 binding protein (C4BP) can inactivate C4b by preventing binding to C2b [59], thereby also limiting C3 convertase formation [55]. In contrast, other soluble factors provide additional checkpoints in complement regulation. For example, although activated fragments of complement will readily hydrolyze in the absence of target attachment [60, 61], inhibitory factor H binds soluble C3b to further reduce off-target effects [62]. C1 inhibitor (C1INH) actually dissociates C1r and C1s from C1q, thereby directly targeting the effector arm of antibody-mediated complement activation [63]. Patients with hereditary angioneurotic edema illustrate the importance of C1INH in complement regulation.

**Figure 2:**
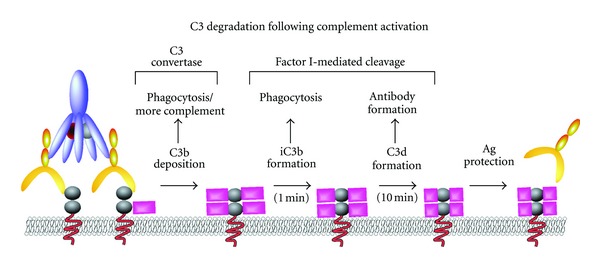
C3 degradation following complement activation. Conversion of C3 to C3b by C3 convertase complex results in rapid deposition of highly reactive C3b on the target surface. Degradation by inhibitory factor I, in concert with other regulatory molecules, results in conversion of C3b to iC3b. Although iC3b retains the capacity of C3b to engage complement receptors as an opsonin, it no longer participates in the production of additional complement. Additional cleavage of iC3b by factor I results in the formation of C3d, which no longer fixes complement or serves as an opsonin, although it may engage B cells and facilitate antibody formation. Decoration of antigen with C3d may result in protection of the antigen from additional antibody binding and subsequent effector function.

Complement degradation also plays a key role in complement regulation. For example, factor I cleaves surface bound C3b and C4b. Although formation of the first cleavage product of C3b, iC3b retains the ability to bind complement receptors, iC3b no longer facilitates complement activation [27]. iC3b can be additionally cleaved by factor I to form C3dg and C3d which are no longer recognized by phagocytosis-inducing complement receptors (Figure 2). Indeed, CD21 serves as the primary receptor for C3dg, where CD21 ligation significantly enhances antibody production against opsonized antigen [45, 64]. Importantly, factor I requires coactivation by several other factors, including factor H, CD35, CD46, and CD55, allowing soluble and membrane inhibitory factors to work in concert to regulate complement activation [55, 65], while also directing complement inactivation toward an individual's own cells. Importantly, several studies recently described additional soluble and membrane bound regulators of complement, adding to the complexity of complement homeostasis [66, 67]. Thus, complement activation reflects a highly regulated pathway responsible for differentially targeting pathogens while protecting an individual's own cells. 

## 5. Factors Influencing Complement Activation

While complement can result in significant pathophysiology during a hemolytic transfusion reaction, not all HTRs result in complement activation [26]. Although the precise factors responsible for dictating whether complement activation will occur remain enigmatic, several factors appears to play a role. Of these factors, the type of antibody and antigen mediating the HTR appears to play the most significant roles in determining the outcome of an incompatible transfusion.

Although different isotypes of antibody fix complement, not all antibodies possess an equivalent capacity to engage this pathway. Appropriate engagement of C1q requires accommodation of two separate CH2 domains on antibody Ig heavy chains [68]. As a result, CH2 availability can influence antibody proclivity for complement fixation. For example, while a single pentameric IgM antibody possesses five separate CH2 domains, an IgG molecule only contains a single domain capable of C1q engagement. In solution, IgM exists as a planar molecule that fails to bind significant C1q, preventing IgM-mediated complement activation in the absence of antigen. However, IgM engagement of antigen induces a conformation change that enables a single IgM to engage C1q and activate complement [69] (Figure 1). In contrast, two IgG molecules must engage distinct antigenic epitopes within 20–30 nm of each other in order to adequately engage C1q and initiate complement [70] (Figure 1). As a result, fewer IgM antibodies must bind antigen to fix equivalent levels of complement as IgG [71]. Equally important, different IgG subclasses possess distinct hinge regions that can differentially impact the conformational flexibility of the heavy chain, thereby also potentially impacting the ability of the CH2 domain to engage complement [72, 73]. Intrinsic differences between subtypes in the CH2 domain may also directly impact C1q engagement [74]. In contrast to IgG and IgM, while IgA may induce complement activation through engagement of the lectin pathway [75], the CH2 domains of IgA, IgD, and IgE possess little affinity for C1q [73]. As a result, IgM and IgG antibodies provide the primary stimulus for classical complement activation.

In addition to intrinsic differences in the ability of Ig isotypes to induce complement activation, a variety of enzymatic and nonenzymatic posttranslational modifications can impact antibody-induced complement activation. For example, posttranslational glycosylation of the Fc domain can also impact the ability of IgG molecules to engage C1q or activate the lectin pathway of complement [76, 77]. Furthermore, nonenzymatic formation of advanced glycation end-products can also affect the ability of antibodies to induce complement activation [78]. Importantly, none of these factors in isolation predicts the ability of an antibody-antigen interaction to result in complement deposition [35]. As a result, the influence of an antibody on complement activation reflects a variety of distinct factors. As polyclonal antibodies typically mediate HTR, the unique composition, affinity, and potential modifications of antibodies within a recipient will impact the likelihood of complement activation during an incompatible transfusion.

In addition to the antibody isotype, the antigen itself appears to independently predict the likelihood of complement activation. For example, ABO(H) HTRs result in robust complement activation and rapid intravascular hemolysis [79]. Similar studies suggest that IgG antibodies against other RBC antigens, including the Kidd antigens (Jk^a^ and Jk^B^) and Fy^a^, appear to induce complement-mediated hemolysis [79, 80]. Other antigens, such as Kell, appear to result in mixed forms of clearance following antibody engagement, utilizing both complement and Fc receptors [81]. It should be noted that many of these studies employed complement deposition, clearance kinetics, or intravascular hemolysis as a surrogate for complement-mediated destruction [79]. Although intravascular hemolysis typically occurs in the presence of complement deposition, it remains to be definitively tested using mammalian animal models whether complement may be required in each of these settings. For example, while some results suggest that complement may be required for both IgM and IgG-induced hemolysis in an animal model of a glycophorin A-mediated HTR [82], a recent study using the same model system suggests that hemolysis occurs independent of Fc receptors or complement [83]. As a result, HTRs reflect complex immunological reactions that need additional investigation in genetically defined animal models to be fully elucidated.

In contrast to ABO(H) HTRs, the Rh(D) antigen, the second most common antigen implicated in HTR, appears to mediate RBC clearance independent of complement. For example, Rh(D) alloimmunized patients who receive incompatible blood display little complement activation, despite several studies demonstrating that similar IgG subclasses facilitate complement activation in other settings [84, 85]. Similarly, administration of Rh immune globulin (RhIG) results in complement-independent hemolysis, likely through Fc-receptor-dependent phagocytosis [86]. Although the Rh antigen complex provides the most compelling example of complement-independent hemolysis, several other antigens, including S and M, also appear to predominately induce HTR independent of complement [79, 87]. 

While the exact mechanism whereby distinct antigens dictate the mode of clearance remains unknown, the density of the antigen likely plays a significant role. Consistent with this, A and B antigens not only represent the most common complement-mediated HTR, but also display the highest antigen density, typically ranging from 700,000 to over a million antigens per cell [88, 89]. In contrast, the Rh(D) antigen typically occurs at approximately 20,000 sites per cell [90]. As a result, the lower density of Rh likely reduces the probability of successful engagement of C1q [73], especially when the antibody composition mediating an HTR primarily reflects the IgG isotype. However, additional antigen factors likely influence this process. For example, although Rh(D) and Kell both reflect low-density antigens [91, 92], Rh mediates complement-independent hemolysis while Kell can engage complement and Fc receptor effector responses [79, 81]. With low-density antigens, antibody-induced clustering likely influences the efficiency of C1q engagement and complement activation [70]. As a result, lateral mobility and location of different antigens within the RBC membrane likely play an important role. Consistent with this, recent studies demonstrate that the cytoplasmic domains of Rh antigens engage RBC cytoskeletal proteins [93], likely limiting the lateral mobility needed to effectively engage C1q. In contrast, Kell possesses a very small cytoplasmic domain with little cytoskeletal attachment [94], thereby potentially enhancing lateral mobility and antibody-induced cluster formation. 

As antithetical antigens occasionally only differ at discrete binding sites, the unique orientation and presentation of antigens may also influence the ability of antibody-antigen complexes to mediate complement fixation. Indeed, several antithetical antigens responsible for HTRs only differ at a single nucleotide polymorphism [95]. As a result, effective antibody-mediated crosslinking and cluster formation may be compromised if each individual molecule only possesses a single epitope. Although some antigens may exist in higher order complexes [96], providing additional epitopes, the availability of the antigenic sites may also be sterically constrained, thus, reducing the overall ability of antibodies to engage antigen. Conversely, carbohydrate A and B antigens display high levels of expression on highly mobile glycolipids and exist as multiple copies on glycoproteins [97], directly facilitating optimal antibody engagement for complement fixation. Taken together, a variety of biochemical features at the antigen level appear to possess the ability to impact antibody-induced complement activation.

Although the type of antibody and antigen can independently impact the probability of a complement-mediated HTR, the type of antigen itself partially dictates the type of antibody generated in response to a particular antigen. Although it remains to be formally examined, previous studies suggest that bacterial flora in the gut that expresses A- and B-like antigens stimulates anti-A and anti-B antibody formation required for an ABO(H) HTR [98, 99]. As these antigens reside on O antigen LPS molecules, they do not typically possess the T cell epitopes required for significant antibody class switching [15]. As a result, anti-A and anti-B antibodies form naturally by the fourth month of life without prior exposure secondary to transfusion and predominately reflect IgM antibodies [15, 100]. 

In contrast to ABO(H) antigens, most antibodies mediating HTR to protein antigens, such as Rh(D), Kell, and Duffy, do not form without prior antigen exposure. Although IgM development during a primary exposure to antigen can actually shorten the lifespan of transfused blood [101, 102], primary antibody responses do not typically result in a routinely detectable HTR. However, during this primary antigen exposure, T cell-mediated class switching results in significant production of IgG. As a result, RBCs transfused into an individual previously immunized against a protein antigen will likely encounter IgG antibodies [103]. Taken together, antibody isotype, antigen density and location, and the modes and mechanisms of antibody stimulation can significantly impact whether complement mediates a HTR. 

## 6. Consequences of Complement Activation

Given optimal antigen and antibody conditions, incompatible transfusion can result in robust activation of complement. Complement activation may not only result in intravascular hemolysis, but complement split products, in particular C5a, can directly impact vascular permeability by activating endothelial cells and inducing significant cytokine production [27, 47]. Thus, complement can directly impact hemodynamic stability following an HTR. Free hemoglobin (Hb) released following complement-mediated hemolysis also induces significant alterations in vascular tone. In addition, Hb may also induce cytokine production, result in significant coagulopathy, and can be directly nephrotoxic [104–108]. As a result, exuberant complement activation following exposure to a large bolus of incompatible RBCs can induce significant pathophysiological changes that may ultimately result in a fatal outcome.

Although rapid complement activation can result in significant mortality, complement-mediated HTRs do not uniformly result in patient death. While several factors, including the volume of incompatible blood and titer of recipient antibody, likely influence patient prognosis following complement-mediated HTR [10, 109], RBC intrinsic factors may also play a role. For example, while ABO(H) HTR may saturate complement inhibitory factors, lower density antigens, such as Kell antigens, likely fix insufficient complement to adequately overcome regulatory pathways, causing arrest of complement activation prior to complete intravascular hemolysis [92]. Indeed, many non-ABO(H) HTRs arrest at the C3b stage of the complement cascade [92]. Although C3b-mediated phagocytic removal may increase cytokine production in these situations [110, 111], limited activation of C5a and reduced release of intravascular Hb also likely limit systemic consequences of complement-mediated HTR. These differences likely contribute to the significantly mortality rate associated with ABO(H) HTR [16]. 

Regulation of complement activation may also influence the rate and magnitude of RBC clearance during a complement-mediated HTR. For example, while C3 retains opsonizing activity following initial factor I-mediated cleavage [27], additional degradation to C3dg and CD3d renders RBCs relatively resistant to C3-mediated removal [112–114] (Figure 2). Consistent with this, complement-mediated HTRs characteristically result in rapid clearance during the first 10 minutes followed by an abrupt change in clearance kinetics that mirror the degradation rate of C3 to C3dg [79, 115, 116]. These results strongly suggest that alterations in clearance at least partially reflect the kinetics of factor I-mediated degradation. Interestingly, once cells accumulate C3dg, they become relatively transparent to the immune system and may circulate with C3dg virtually undetected by phagocytic cells [115–117] (Figure 2). In addition, ongoing degradation of complement may actually result in release of phagocyte-engaged cells, as several studies demonstrate that RBC can reappear hours following a complement-mediated HTR [110, 118]. 

A variety of additional variables can influence the extent and consequences of complement activation following an HTR. For example, although stochastic competition between activating and inhibitory stimuli likely dictates which cells will resist complement-mediated lysis, recent studies suggest that the activity of complement inhibitory proteins may be altered as RBCs age. Indeed, accumulation of advanced glycation end products on complement inhibitory proteins appears to directly diminish their function, strongly suggesting that older RBCs may be more sensitive to complement-mediated hemolysis [119]. Furthermore, the age of the patient may also impact the extent of complement-mediated hemolysis, as pediatric patients possess a less mature complement inhibitory apparatus than adults [120]. Thus a variety of cell-specific factors may also independently regulate the consequences of complement activation.

While many modifications or alterations in the expression of complement inhibitor proteins can impact RBC sensitivity to complement-mediated hemolysis, additional alterations affecting general RBC physiology may also impact complement sensitivity. In addition to hemolyzing transfused cells during HTR, occasionally patients will hemolyze their own cells, a phenomenon called bystander hemolysis [121]. Although bystander hemolysis may theoretically occur in any patient [122], individuals with sickle cell disease appear to be particularly prone [121]. Interestingly, several studies suggest that sickle cells may be intrinsically sensitive to complement-mediated lysis, suggesting that complement may in part be responsible [123, 124]. However, as several antigens that may not activate complement, such as Duffy, can also induce bystander hemolysis [125], the actual role of complement in the pathophysiology of this process remains largely unknown. In addition, complement activation may also be involved in non-hemolytic transfusion reactions, such as transfusion-related acute lung injury (TRALI) [126], another leading cause of transfusion-related mortality [127]. Indeed, recent studies suggest that, in addition to anti-HLA antibodies, anti-neutrophil antibodies, and other factors [127], differences in complement may also contribute to the gender influence of donor plasma on the development of TRALI [128].

## 7. Possible Complement-Targeted Therapeutic Interventions

Once an HTR is suspected, immediate intervention takes place in the form of stopping the transfusion and initiating intravenous fluids in an effort to achieve hemodynamic stability and protect the kidneys from the nephrotoxic effects of hemoglobin. However, no intervention in routine clinical practice specifically targets the HTR itself. As some patients, particularly patients with paroxysmal nocturnal hemoglobinuria, suffer from chronic hemolysis secondary to a deficiency in CD55 and CD59, a number of complement inhibitors have been developed in an effort to treat this and other complement-related disorders. While many inhibitors exist [129–135], only a few inhibitors have been evaluated using animal models of HTRs and no inhibitors have been formally examined clinically in an HTR setting. Injection of a soluble form of CR1 significantly attenuates complement activation following transfusion and increases posttransfusion survival of incompatible blood. Similar results were obtained using small molecule inhibitors of complement or smaller fragments of CR1 [136, 137]. Specific targeting of complement inhibition to RBCs through antibody-DAF chimeras may also inhibit complement-induced hemolysis [138]. Additional experimental models also suggest that intravenous immune globulin (IVIG) can inhibit complement-mediated HTRs [139, 140]. Although many of these inhibitors demonstrate promise in animal models, other complement inhibitors, including the C5 inhibitor, eculizumab [141], which are currently available for clinical use in other settings, need to be formally examined in the setting of HTR. As a result, future studies need to explore the potential role of complement inhibition as a prophylactic agent in the event that antigen-compatible blood may not be available or as therapy following an unfortunate HTR.

Although reengagement of antibody with antigen would be expected to provide a continuous supply of active complement, RBCs represent the only cells in the human body that do not resynthesize protein following maturation. As a result, whatever antigen exists on an RBC represents the only antigen that RBC will possess for the duration of its lifetime. While this may not alter basic antibody-antigen interactions, as C3 molecules attach to target membrane through covalent association, previous studies suggest that deposition of complement on antigens can sterically or directly mask targets from additional antibody binding [115, 116, 142–144] (Figure 2). As a result, once antigen masking and complement degradation are complete, cells become relatively transparent to the effector mechanism of hemolysis. These studies suggest that deposition and inactivation of complement *in vitro *may allow complement-mediated masking of antigen prior to transfusion in an effort to generate compatible RBCs for highly immunized patients (Figure 2). While these possibilities are intriguing, more studies are needed utilizing defined animal models to determine whether complement-mediated RBC manipulation might enable transfusion across immunological barriers.

## 8. Conclusions

HTRs historically provided significant insight into the role of complement as an antibody effector system [79]. Fortunately for patients, extreme vigilance exercised by transfusion medicine services has drastically reduced the rate of hemolytic transfusions [9]. As a result, most detailed studies examining HTRs predate the exponential increase in genetically manipulated animal models designed to appreciate subtle, yet critical, factors responsible for complement regulation *in vivo* [145–147]. Furthermore, until recently, no mammalian models existed to study clinically relevant RBC antigens in the setting of HTR [82, 148–151]. Indeed, despite Landsteiner's discovery of ABO(H) antigens over a century ago, no animal model currently exits to formally examine the role of complement or other factors in ABO(H) HTRs. Despite the relatively decreased incidence of mistransfusion and HTRs, the risk still remains. In 2011, HTR represented the second leading cause of transfusion-associated mortality reported to the FDA [152]. Thus, additional models need to be developed with a renewed focus on the mechanisms responsible for complement-dependent and complement-independent mechanisms of RBC hemolysis. These future studies possess the potential to provide rational approaches to not only develop therapeutic interventions in the unfortunate event that an HTR occurs, but to also extend the scope of immunological compatibility by manipulating complement to render otherwise incompatible transfusions compatible. Future work will likely provide significant insight into complement activation and regulation with the potential to significantly impact patient care.
